# Antiaggregation Potential of *Padina gymnospora* against the Toxic Alzheimer’s Beta-Amyloid Peptide _25–35_ and Cholinesterase Inhibitory Property of Its Bioactive Compounds

**DOI:** 10.1371/journal.pone.0141708

**Published:** 2015-11-04

**Authors:** Balakrishnan Shanmuganathan, Dicson Sheeja Malar, Sethuraman Sathya, Kasi Pandima Devi

**Affiliations:** Department of Biotechnology, Alagappa University (Science campus), Karaikudi- 630 004, Tamil Nadu, India; University of Lancaster, UNITED KINGDOM

## Abstract

Inhibition of β-amyloid (Aβ) aggregation in the cerebral cortex of the brain is a promising therapeutic and defensive strategy in identification of disease modifying agents for Alzheimer’s disease (AD). Since natural products are considered as the current alternative trend for the discovery of AD drugs, the present study aims at the evaluation of anti-amyloidogenic potential of the marine seaweed *Padina gymnospora*. Prevention of aggregation and disaggregation of the mature fibril formation of Aβ _25–35_ by acetone extracts of *P*. *gymnospora* (ACTPG) was evaluated in two phases by Thioflavin T assay. The results were further confirmed by confocal laser scanning microscopy (CLSM) analysis and Fourier transform infrared (FTIR) spectroscopic analysis. The results of antiaggregation and disaggregation assay showed that the increase in fluorescence intensity of aggregated Aβ and the co-treatment of ACTPG (250 μg/ml) with Aβ _25–35_, an extensive decrease in the fluorescence intensity was observed in both phases, which suggests that ACTPG prevents the oligomers formation and disaggregation of mature fibrils. In addition, ACTPG was subjected to column chromatography and the bioactivity was screened based on the cholinesterase inhibitory activity. Finally, the active fraction was subjected to LC-MS/MS analysis for the identification of bioactive compounds. Overall, the results suggest that the bioactive compound alpha bisabolol present in the alga might be responsible for the observed cholinesterase inhibition with the IC50 value < 10 μg/ml for both AChE and BuChE when compared to standard drug donepezil (IC50 value < 6 μg/ml) and support its use for the treatment of neurological disorders.

## Introduction

Alzheimer’s disease (AD), an age related devastating neurodegenerative disorder results in cognitive impairments such as decision-making, language and behavioral activities. In recent years, several etiological factors have been linked to AD pathology, which include Aβ peptide (Aβ) and τ protein aggregation, metal ion accumulation, oxidative stress and reduction in cholinergic neurotransmission [1, 2, 3]. Among the factors, the most hopeful approach is the usage of antiamyloidogenic agents which could prevent aggregation of Aβ peptides. A senile plaque (SPs) is one of the most important neuropathological hallmarks of AD, and the major constituent of SPs is beta-amyloid (Aβ), which accumulates in the outer surface of the neurons. Aβ is the product of proteolytic cleavage of the amyloid precursor protein (APP) by β-secretase and γ-secretase [4]. Particularly, the accrual level of Aβ oligomers correlates with the severity of cognitive impairment in patients with AD and play a vital role in AD pathology. Many clinicopathological studies have demonstrated that the deposition of insoluble Aβ produces the aggregation of amyloid fibrils in brain parenchyma and cerebral blood vessels, which is one of the major hallmarks of AD [5]. Extracellular aggregates of Aβ was observed in AD patients, the most predominant one is Aβ _1–40_ or Aβ _1–42_; however, they also contain peptides with shorter sequences such as Aβ _25–35_ fragment containing a stretch of 11 full length amino acid residues. It forms itself β-sheet structure and produces similar effects to those produced by its parent sequence and has been found to be biologically active conferring toxicity to neurons [6]. Pike et al demonstrated that Aβ peptides that exist in an aggregated state are directly toxic to cultured neurons, while insoluble Aβ peptides lack direct toxicity. Aβ _25–35_ peptide, the biologically active fragment includes both a hydrophilic domain (25–28) that contains a putative β -turn site and a hydrophobic domain (29–35) essential for stable aggregation [7]. Some neuropathological studies have found that low-molecular weight of amyloid oligomers subsequently give rise to high-molecular weight amyloid oligomer, termed as soluble aggregation intermediates. After soluble aggregation formation, the intermediate further aggregates to form fibrils along with hyperphosphorylated tau protein, which forms senile plaques in the hippocampal region of the brain [8]. It is related to the level of cognitive impairment in AD and local distribution of amyloid burden is often correlated with changes in the cognitive functions [9, 10].

Several reports suggest that huge number of environmental factors along with the inherent properties of Aβ, collectively results in the deposition of Aβ aggregates. Although the involvement of its molecular mechanism in development and progression of AD is not clear, a critical role of Aβ is universally accepted [11]. Apart from forming plaques, the Aβ oligomers are also more effective as neurotoxins that cause disruption of neuronal synaptic plasticity, which suggest that, inhibition of Aβ oligomerisation might lead to novel therapeutic method for AD treatment [12, 13] Recent reports illustrated that oxidative stress also plays a significant role in AD pathogenesis and Aβ peptides have been proposed as a source of oxidative stress. Oxidative stress induced by Aβ leads to increased oxidative modification of proteins and lipids which in turn leads to impaired cellular function, cell death and consequently cognitive decline and AD pathology [14].

In recent times, bioactive compounds derived from natural sources are attracting increasing awareness in the search of new drugs for AD treatment. Seaweeds or marine algae possess essential polysaccharides with huge number of secondary metabolites that might have bioactive properties for their use as foods, pharmaceuticals and cosmeceuticals. These secondary metabolites from seaweeds have potentially significant therapeutic values and provide a great variety of biological compounds for sampling in the phase of drug discovery and development [15]. Based on this, the current study is mainly focused on the marine brown seaweed *P*. *gymnospora* for the detection of anti-amyloidogenic agents. *P*. *gymnospora* is known to possess a variety of valuable medicinal properties such as anti-cancer, anti-coagulant, anti-bacterial, anti-angiogenic and anti-adhesive activities [9, 16, 17, 18, 19]. Though *P*. *gymnospora* has been reported for its therapeutic potential to cure other human ailments, reports on its possible neuroprotective potential is not available. Consequently the objective of the work is designed to assess the anti-aggregation and disaggregation property of brown seaweed *P*. *gymnospora* against Alzheimer’s beta-amyloid peptide 25–35, which may provide a lead for the identification of novel therapeutic compounds with an ability to combat AD. In addition to pharmacological agents, extracts derived from natural source have bioactive potential and are used as a good candidate for AD treatment. Initial screening of the seaweed under study *P*. *gymnospora* which is densely inhabited in the Gulf of Mannar region, for anti-AD properties revealed that the acetone extract of *P*. *gymnospora* (ACTPG) possesses excellent antioxidant activity and cholinesterase inhibitory activity *in vitro*. The promising results of the preliminary studies prompted us to further extend the study by verifying its anti-amyloidogenic activities. The present study showed an insight into the bioactive compounds present in the alga, which might be responsible for the observed cholinesterase inhibition. The current study undoubtedly shows the antiaggregation and disaggregation potentials of *P*. *gymnospora*, which has also provided a novel bioactive compound Alpha-bisabolol, which can be used to combat AD.

## Materials and Methods

### Chemicals

AChE from Electric eel (Type-VI-S, EC 3.1.1.7, Sigma) and BuChE from horse serum (EC 3.1.1.8, MP Biomedicals) were used as enzyme source, acetylthiocholine iodide (ATCI) and butyrylthiocholine iodide (BTCI) (Himedia laboratories, Mumbai, India) were used as substrates.5,5′-Dithio-bis (2-nitrobenzoic) acid (DTNB) was procured from Himedia laboratories, Mumbai, India. Aβ _25–35_ peptide was purchased from GenScript USA Inc. Thioflavin T (ThT) and L-carnosine was obtained from Sigma Aldrich Co. LLC. Alpha-bisabolol and caffeine were obtained from Alfa Aesar, UK. All the other reagents and chemicals used were of analytical grade.

### Preparation of seaweed extract

#### Collection and processing of seaweed samples

Seaweed (*Padina gymnospora*) was collected from the intertidal region of Gulf of Mannar region, Tamil Nadu, and the species was identified according to the reference [20, 21]. The seaweed which was washed off the shore was collected for the experiments, for which permission is not required. We confirm that the field studies did not involve endangered or protected species. According to the method of Ratnasooriya et al the collected seaweeds were processed to remove the attached specimens on its surface [22]. The samples were then washed with tap water, distilled water and then with 70% alcohol to remove the adhered microflora. The processed seaweed was dried under shade and stored in an airtight zip-lock container. For extraction, 100 g of dried seaweed was packed in a Soxhlet apparatus by using Whatman No. 1 filter paper and extracted with respective solvents (l000 ml). The process of successive extraction was carried out for 6 h using different solvents (ranging from non polar to polar). After extraction, all the extracts were filtered by using Whatman No. 1 filter paper and subjected to dryness under reduced pressure in a vacuum desiccator. The dried extract was dissolved in distilled water containing less than 0.02% of methanol or Tween 20 as solvents and used for further analysis. The extraction procedures were done at a temperature less than 40°C to avoid thermal degradation of the compounds.

### Antiaggregation and disaggregation assays

According to the method of Ramesh et al the anti-aggregation and disaggregation property of *P*. *gymnospora* was evaluated in two different phases [12]. In phase I, the inhibition of aggregate formation was assessed by using oligomers. Consequently, in Phase II the disaggregation of the pre-formed fibrils were determined. Freshly prepared Aβ _25–35_ (100 μM) monomer was incubated in Tris-HCl buffer pH 7.4 at 37°C for 20 h. However, after 20 h, an increase in fluorescence intensity was observed which represents the formation of oligomers. The acetone extract of *P*. *gymnospora* (ACTPG) (250 and 500 μg/ml) was added to the oligomers and subjected to incubation at 48 h for dose fixation. After fixing the optimum concentration of ACTPG, the Aβ _25–35_ oligomers were incubated with/without ACTPG (250 μg/ml) for 20 h, 48 h, 96 h and 9 days respectively. In phase I, the freshly prepared Aβ _25–35_ (100 μM) was incubated with ACTPG (250 μg/ml), and the aggregation pattern was evaluated by ThT assay with the aliquots taken from the incubation mixture at 20 h and 48 h respectively. Whereas in phase II, the freshly prepared Aβ _25–35_ (100 μM) was incubated for 96 hours, for the formation of mature fibrils. After that, ACTPG (250 μg/ml) was co-incubated with the pre-formed fibrils, and the disaggregation pattern was evaluated by taking the aliquots of incubation mixture at 96 h and 9 days using Thioflavin T assay using the standard Galantamine (50μM) as a positive control. Additionally, microfluorescence assay and Fourier transform infrared analysis (FT-IR) were employed to further confirm the anti-aggregation and disaggregation property of *P*. *gymnospora*.

#### Thioflavin T assay

Thioflavin T-induced fluorescence changes were measured to quantify amyloid fibril formation by spectrofluorimeter (Microplate Analyzer) as described by [23]. Aliquots (20 μl) from the incubation mixtures of both the phases were added to 50 mM glycine—NaOH buffer (pH 8.5) containing 5 mM Thioflavin T in a final volume of 1 ml. Each sample was analyzed in triplicates, and the fluorescence intensities were measured at 450 nm (excitation) and 485 nm (emission) under time resolved fluorescence mode in Spectramax M3 reader (Molecular Devices). The background fluorescence emitted by ThT was subtracted from the values of all the samples.

#### Microfluorescence assay

About 2.5 μl aliquot of the fibrillated Aβ _25–35_ peptide (100 μM) sample was diluted (1:2) with 5 μM thioflavin T in 50 mM glycine-NaOH buffer (pH 8.5) and transferred onto a slide. Fluorescent signals (488 nm) were then visualized by the Confocal Laser Microscope System (CLSM FV300, Olympus, Tokyo, Japan) and processed by Adobe Photoshop (Adobe Systems, Mountain View, CA, USA). The fluorescence intensity was visualized in each of three random fields of the sample.

#### FT-IR

Alterations in the structural characteristics of Aβ _25–35_ peptide was analyzed through FTIR and the spectra were recorded in Nicolet iS5 FT-IR Spectrometer (Thermo Scientific, Marietta, GA, USA) using OMNIC software [24]. The Aβ _25–35_ peptide (100 μM) were incubated with/without ACTPG (250 μg/ml) for 20 h, 48 h, 96 h and 9 days respectively. For analysis, a small portion of the incubated sample mixed with potassium bromide was scanned from 4,000–400 cm^−1^ with a spectral resolution of 4 cm^−1^.

#### Purification of ACTPG using column chromatography

Freeze dried bioactive ACTPG (10 gm) was impregnated with silica gel 60–120 mesh and loaded onto silica gel (100 × 35 mm) column. The column was thoroughly washed with hexane and eluted with linear gradient of solvent of n-hexane, CLFM, EA, ACT, MET and WAT with increasing polarity. Fractions were collected, freeze dried and the bioactivity was screened based on antioxidant and cholinesterase inhibitory activity.

#### LC-MS/MS analysis of ACTPG

Freeze dried bioactive ACTPG samples were subjected to LC-MS/MS by using reverse phase C-18 column. The compounds were isolated with an acetonitrile/water (50:50) at a flow of 0.2 mL min-1. The column temperature was set to 25°C during all the running and the injection volume was 15 μl. Mass spectra was performed with software (HYSTAR+ otof control bruker) and the spectra was recorded in LC-MS/MS (Bruker microtof III). Electron spray ionization (positive mode) with full scan was used for analyzing the compound. The identity of the compound and its molecular mass from 50–3000 m/z was confirmed by comparing their m/z ratio with those on the stored library.

#### AChE and BuChE inhibitory assay for identified compounds

ChE inhibitory activities were measured using a modified 96-well microplate assay developed by [25]. Briefly, AChE/BuChE (10 μl at 10 U/ml) solution was incubated with various concentrations of three different major compounds of *P*. *gymnospora* such as L-Carnosine, alpha bisabolol and Caffeine in 0.05 M Tris-HCl buffer (pH 8.0/7.4) for 45 min at room temperature (RT). After incubation, 125 μl of 3 mM DTNB was added and the total volume was made up to 300 μl with Tris- HCl buffer (pH 8.0/7.4). Enzyme activity was initiated by the addition of 50 μl of 15 mm ATCI/BTCI. The hydrolysis of ATCI and BTCI was monitored by the formation of the yellow 5-thio-2-nitrobenzoate anion at 405 nm for 3 min using Model 680 Microplate Reader (Bio Rad, California). Donepezil (10–50 μg/ml), the standard anti-cholinesterase drug was used as reference. AChE activity was expressed as U/mg of protein (1U = μmol of thiocholine iodide formed/min/mg protein). The experiments were done in triplicate and the results are represented as % of inhibition, which was calculated using the molar extinction coefficient of DTNB (ε_λ_ = 13,600 M^-1^ cm^-1^). Percentage of inhibition of AChE/BuChE was determined by comparison of rates of reaction of samples relative to the blank (Tris-HCl buffer) using the formula, where SA is the specific activity.

% of inhibition = [(SA of control − SA of test sample) / SA of control]*100

### Statistical analysis

Statistical analysis was performed using SPSS 17.0 software package. The results of all the experiments were represented as mean ± SD of triplicates employed. Analysis of variance was performed by one-way ANOVA. Significant differences between control and treated groups were determined by Duncan’s multiple range tests and *p* value < 0.1 were regarded as significant. The IC_50_ value was determined for different bioactive compounds present in *P*. *gymnospora* using Probit analysis.

## Result and Discussion

### Antiaggregation and disaggregation property of Aβ _25–35_ by acetone extract of *P*. *gymnospora* (ACTPG)

The freshly prepared Aβ _25–35_ (100 μM) monomer was incubated in Tris-HCl buffer pH 7.4 at 37°C for 20 h to form oligomers. The ACTPG (250 and 500 μg/ml) was added to oligomers and subjected to incubation at 48 h for dose fixation. After fixing the optimum concentration of ACTPG, the Aβ _25–35_ oligomers were incubated with/without ACTPG (250 μg/ml) at different time intervals and the aliquots of 20 μl were drawn from the incubation mixture at 20 h, 48 h, 96 h and 9d respectively. Fujiwara et al reported that crude extracts of plants rich in polyphenolic content exhibits potent anti-amyloidogenic properties [26]. In 2012, Vauzour has demonstrated that plant based compounds are rich in polyphenol such as curcumin, rosmarinic acid, tannic acid, catechin and quercetin have an ability to inhibit the Aβ fibril formation *in vitro*[27]. Based on these observations, the present study was carried out to assess the inhibition of fibril formation by ACTPG under *in vitro* conditions using Thioflavin T assay and the results were further validated through confocal microscopy study and Fourier Transform Infrared (FTIR) spectroscopic analysis.

Many chemical ligands have been developed as Aβ aggregation inhibitors in recent years including Curcumin and scyllo-inositol [13, 28] but very few have progressed to clinical trials. Considering this disappointing situation, it is appropriate to search for alternative Aβ aggregation inhibitors among natural products. Some fungal and plant extracts have remarkable anti-AD activities *in vitro* and *in vivo* due to the inhibition of Aβ aggregation [13]. Durairajan et al reported that Salvianolic acid B (Sal B) derived from *Salvia miltiorrhiza*, a Chinese herbal medicine commonly used for the treatment of cardiovascular and cerebrovascular disorders, has been found to possess inhibitory effect on Aβ fibrillation and disaggregation of fibrils [29]. Such studies justify further research on natural products, which could identify lead compounds for AD treatment.

#### Thioflavin T assay

**Fig 1:**
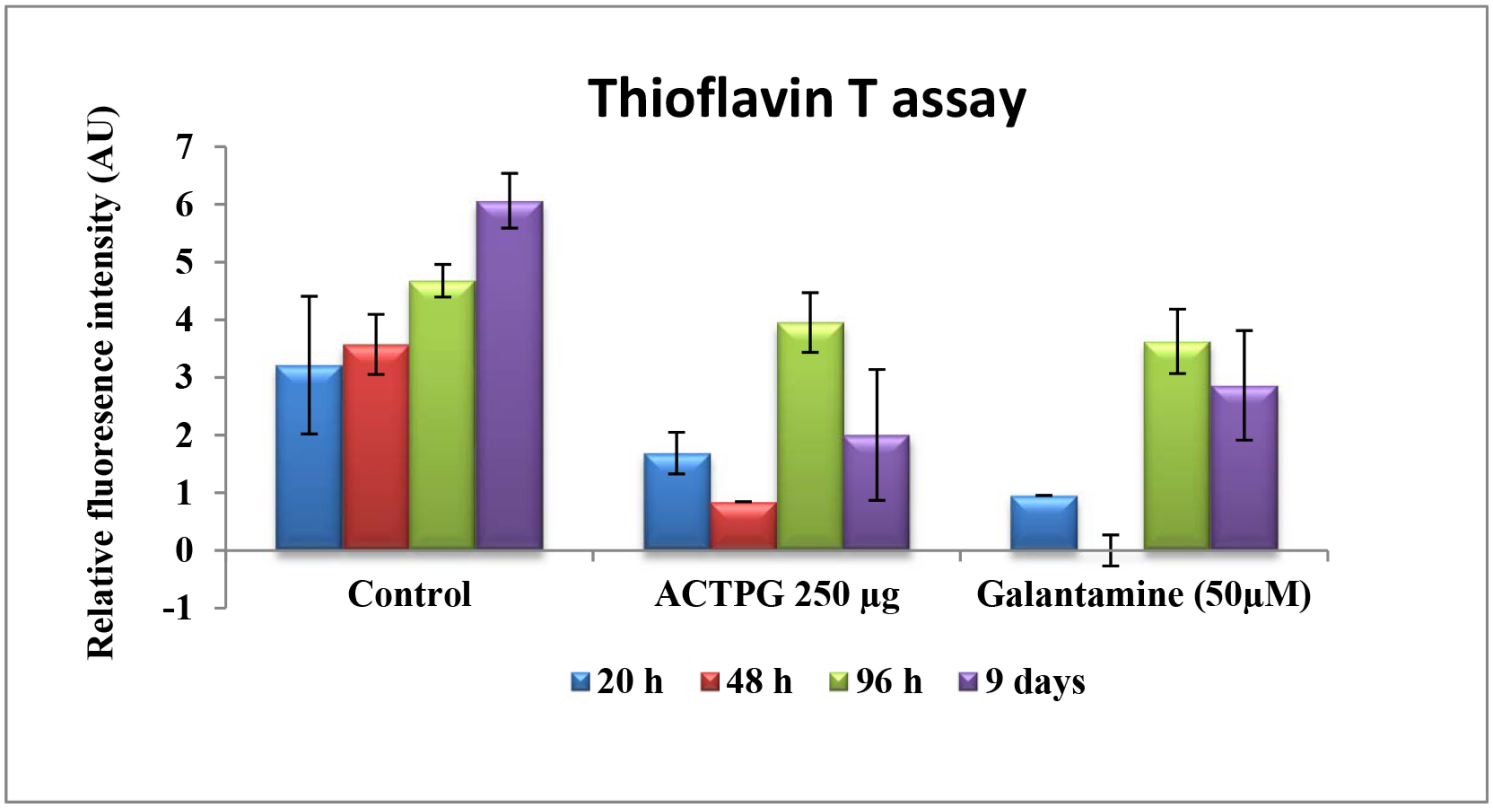
Effects of ACTPG on Aβ _25–35_ fibrillogenesis. Analysis of the deposited amyloid aggregates as assessed by thioflavin T (ThT) assays in Phase I (20, 48 h) & II (96 h & 9days). Quantitative effects of ACTPG on Aβ _25–35_ fibrillogenesis by ThT assay showing the bar diagram of thioflavin-T fluorescence of Aβ _25–35_ in the presence and absence of ACTPG. The emission spectrum of ACTPG and galantamine alone was subtracted, and emission data of peptide dispersions were normalized. Values are expressed as Mean ± SD (n = 3).

Thioflavin T assay was performed to assess the antiaggregation and disaggregation property of Aβ peptide [23]. The results of Thioflavin- T fluorescence assay was performed in two different phases at different time intervals (20 h, 48 h, 96 h and 9d) are shown in Fig 1. The results show that incubation of Aβ peptide at 37°C for 20 h the fluorescence intensity was less (3.21 ± 1.19 AU); however, at 48 h of incubation, the fluorescence intensity is slightly increased (3.57 ± 0.52 AU), which indicates the commencement of aggregation. Attractively, upon co-treatment of ACTPG (250μg/ml) with Aβ _25–35_, an extensive decrease in the fluorescence intensity (1.68 ± 0.3 AU, 0.84 AU) was observed, which indicates that the extract prevents the formation of fibrils from oligomers in Phase I. In addition, ACTPG disaggregates the pre-formed fibrils in Phase II. A prominent increase in fluorescence from 96 h to 9 days was observed in Aβ _25–35_ alone, and the observed fluorescence intensity was 4.67 ± 0.28 AU, 6.06 ± 0.47 AU respectively. The significant decrease in fluorescence intensity in ACTPG (3.95 ± 0.51 AU, 2.00 ± 1.13AU) and Galantamine (3.62 ± 0.55 AU, 2.86 ± 0.94 AU) treated groups indicate that the ACTPG and galantamine reduced β-sheet by preventing the fibrillation.

#### Microfluorescence assay

**Fig 2:**
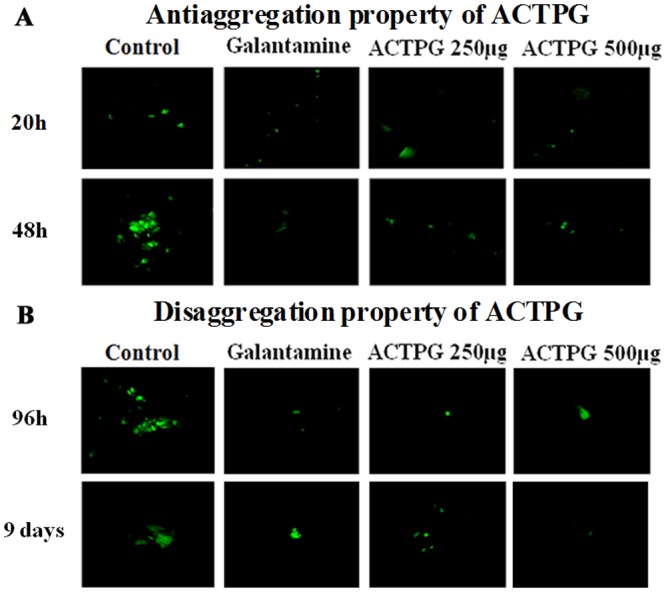
Effects of ACTPG on antiaggregation and disaggregation property of Aβ _25–35_. Analysis of the deposited amyloid aggregates as assessed by Confocal Laser Microscope System (CLSM FV300, Olympus, Tokyo, Japan) in (A) Phase I (20, 48 h) & (B) Phase II (96 h & 9days) and processed by Adobe Photoshop (Adobe Systems, Mountain View, CA, USA). The fluorescence intensity was visualized in each of three random fields of the sample. Confocal microscopic image represents a view of deposited Aβ _25–35_ amyloid aggregates, with representative fibrils from Aβ _25–35_ samples (control) and Aβ _25–35_ samples incubated with the presence and absence of ACTPG and galantamine.

In microfluorescence assay, an increase in the fluorescence was observed in control group (Aβ _25–35_) at 20 h, 48 h, 96 h and 9 days indicating the fibrillar formation. The results of Thioflavin T assay was further validated by CLSM analysis. The CLSM analysis reveals that the peptide aggregation starts after 20 hours and it increases rapidly at 48 hours with an enhanced fluorescence emission in control group. Co-treatment with ACTPG possessed potent anti-aggregatory property of Aβ 25–35 peptide, as observed by the decreased fluorescence intensities (Fig 2A). Prevention of fibril formation from the oligomers was visible by the smaller non aggregated form of peptides even after incubating at 96 hours. A complete breakdown of the mature fibrils into smaller peptides was visible, and the extract was able to hold the destabilization potential even after 9 days of incubation (Fig 2B). CLSM system images (Fig 2A and 2B) confirmed that the aggregated forms of Aβ _25–35_ were visible in control group at different time intervals, and furthermore, the inhibition of aggregation and disaggregation Aβ fibrils was observed in extract-treated group. A similar inhibitory effect on Aβ _25–35_ aggregation was observed in galantamine-treated group. Therefore, the results of these experiments indicate that ACTPG could be able to prevent fibril formation and also to disaggregate Aβ fibrils even after the aggregation process was initiated. However, a marked decrease in fluorescence was observed in ACTPG and Galantamine treated groups, which indicates the antiaggregation and disaggregation property of ACTPG on Aβ fibrils.

#### FTIR spectroscopic analysis of ACTPG on Aβ _25–35_ peptide

**Fig 3:**
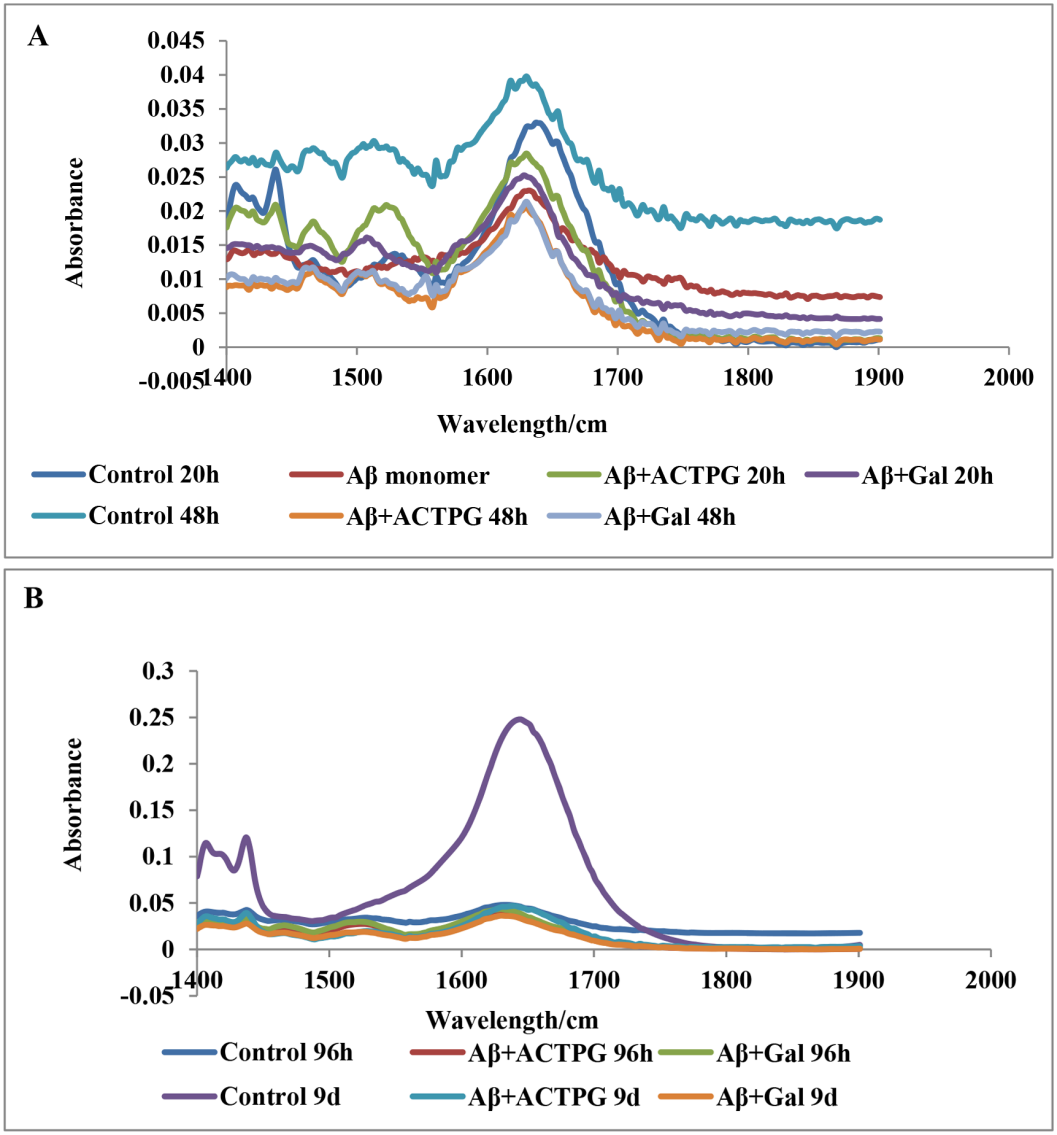
**(A)Inhibitory effects of ACTPG on the transformation of secondary structure of Aβ**_**25–35**_ **by FT-IR.** The secondary structure of Aβ_25–35_ was followed by observing the amide I and amide II regions by FT-IR at different time intervals in phase I, which are the well-known vibrational IR bands particularly used to reveal the conformational changes of peptides and proteins. FTIR spectrum of Aβ_25–35_ (100 μM) exhibited maximum absorbance at 1600 cm^-1^, at 20 and 48 h in phase I, while the ACTPG treated groups showed reduction in absorbance similar to the standard galantamine, which illustrates that ACTPG prevents the formation of fibrils from oligomers. **(B) Inhibitory effects of ACTPG on Aβ**_**25–35**_ **peptide by FT-IR.** The secondary structure of Aβ _25–35_ was followed by observing the amide I and amide II regions by FT-IR at different time intervals in phase II. which are the well-known vibrational IR bands particularly used to reveal the conformational changes of peptides and proteins. In phase II (96h and 9days) FTIR spectrum exhibited increase in absorbance at 1600 cm^-1^ in Aβ treated groups, whereas no such shift was observed in ACTPG-treated groups, which indicates the disaggregation property of ACTPG.

The anti-aggregation and disaggregation potential of ACTPG was further evaluated through FTIR analysis. Among the different spectroscopic techniques, IR spectroscopy has been widely used for the structural characterization and analysis of peptides and proteins [24]. Although there are different IR absorption bands, the amide I (1600–1690 cm^-1^ C = O stretching) and amide II (1480–1575 cm^-1^ CN stretching, NH bending) regions are the well-known vibrational IR bands, which are used particularly to reveal the conformational changes of peptides and proteins. Aβ_25–35_ exhibited maximum absorbance at 1600 cm^-1^, at 20 and 48 h, while the extract treated groups showed reduction in absorbance similar to the standard galantamine, which illustrates that extract prevents the formation of fibrils from oligomers. In addition, at 96 h and 9 days, increase in absorbance was observed at 1600 cm^-1^ in Aβ treated groups, whereas no such shift was observed in extract-treated groups, which indicates the disaggregation property of extract. Results of FTIR analysis (Fig 3) suggests that ACTPG restores the alteration in the conformational state of Aβ _25–35_ in 20 h, 48 h, 96 h and 9days by preventing the aggregation process.

#### Bioactivity guided fractionation of ACTPG using column chromatography

**Fig 4:**
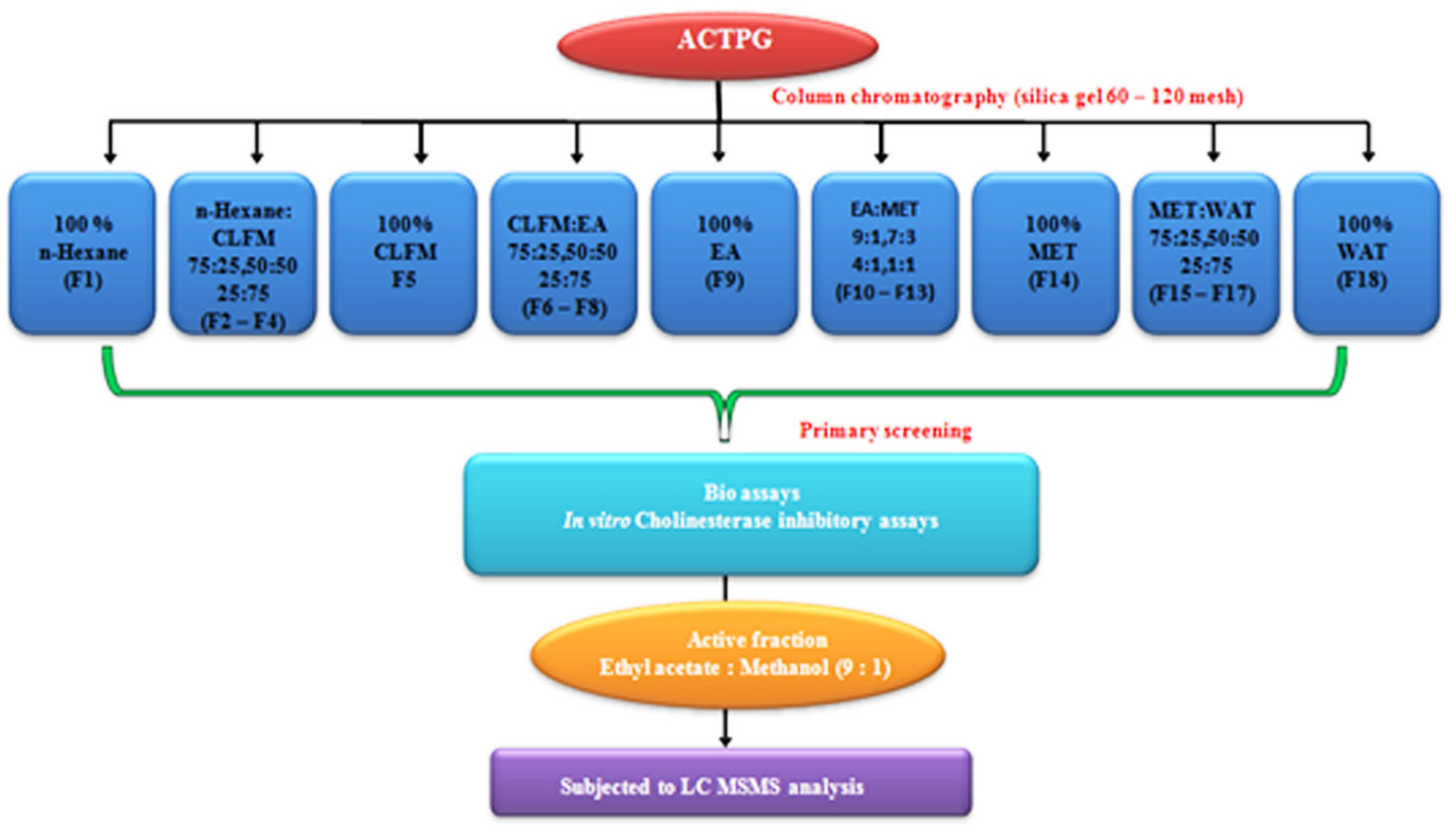
Bioactivity guided fractionation of ACTPG. Bioactive fractions were eluted with linear gradient of solvent with increasing polarity from n-hexane to water and the 18 fractions (F1-F18) were collected and subjected to cholinesterase inhibition assay.

**Fig 5:**
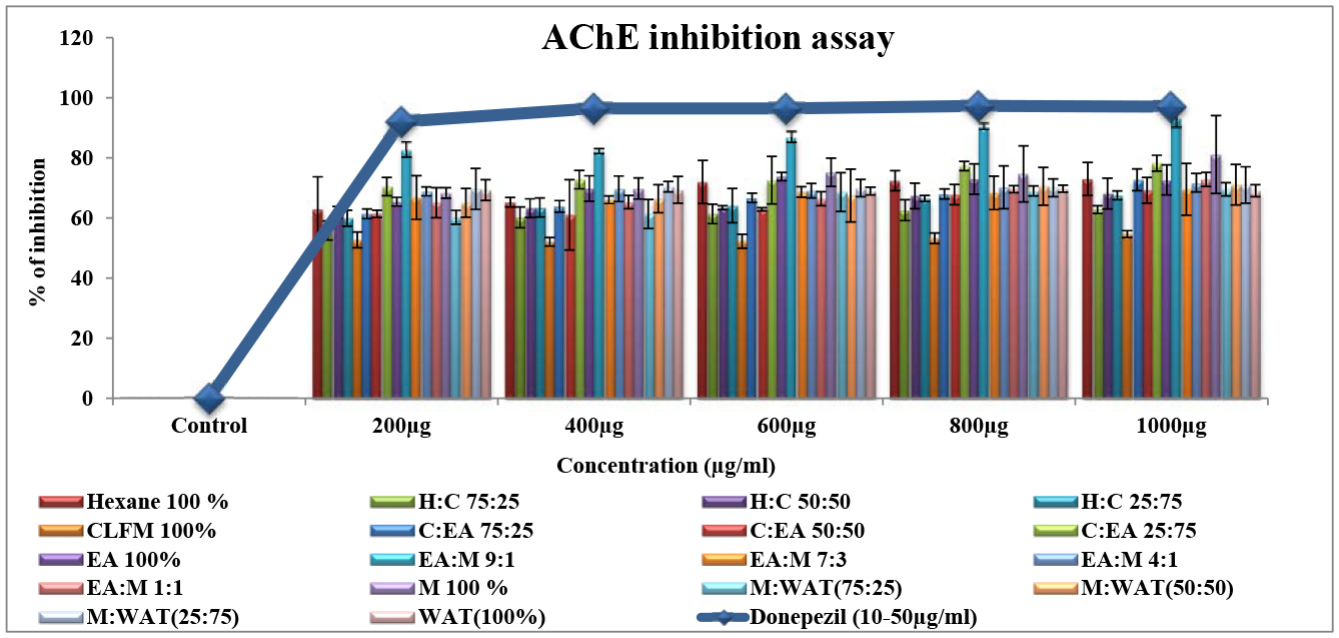
AChE inhibitory activity of the column fractions (F1-F18). The eluted fractions (F1-F18) were subjected to AChE inhibitory assay. Among the fractions, F10 (EA: MET-9:1) showed highest inhibitory activity (93%) against AChE, but similar when compared to positive control donepezil (97%). Values are expressed as Mean ± SD (n = 3).

**Fig 6:**
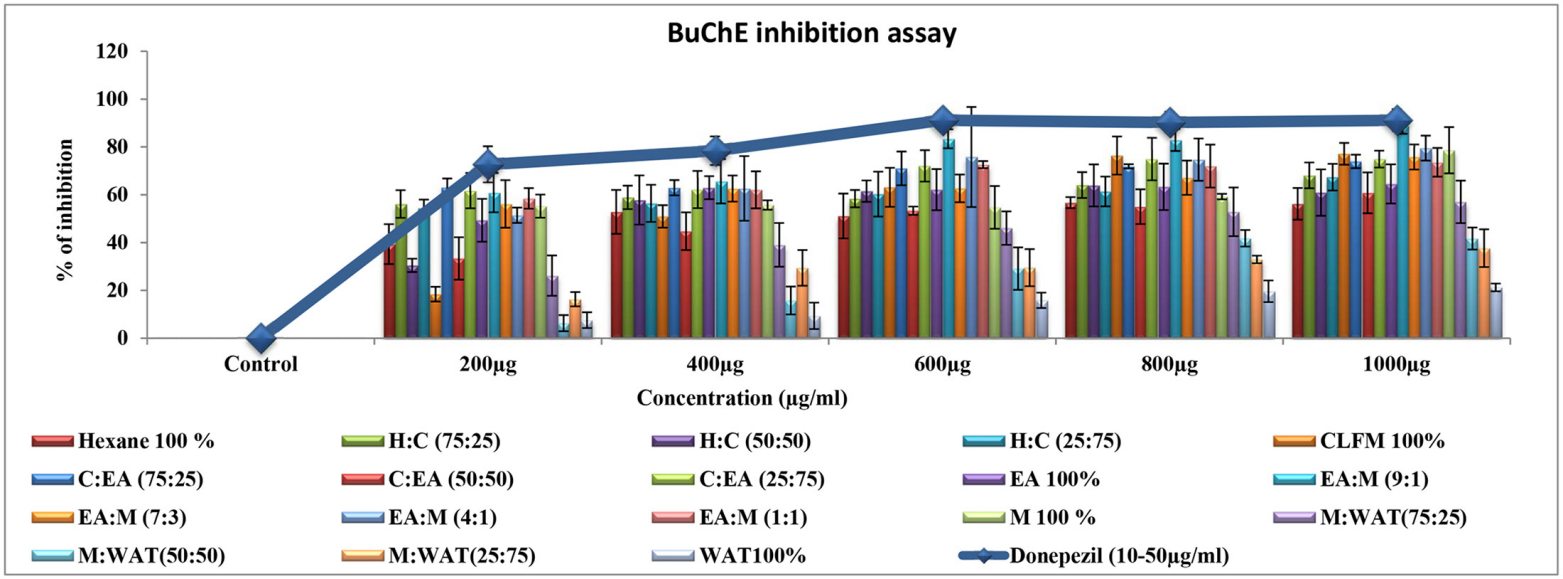
BuChE inhibitory activity of the column fractions (F1-F18). The eluted fractions (F1-F18) were subjected to BuChE inhibitory assay. Among the fractions, F10 (EA: MET-9:1) showed highest inhibitory activity (88%) against BuChE, but similar when compared to positive control donepezil (91%). Values are expressed as Mean ± SD (n = 3).

Bioactive fractions were eluted with linear gradient of solvent with increasing polarity and 18 fractions (F1-F18) were collected based on polarity from n-hexane to water as represented in Fig 4. Eluted fractions were subjected to cholinesterase inhibitory assays. Among the different fractions F10 showed potent cholinesterase inhibitory activity. Results of AChE and BuChE inhibitory activity were illustrated in Figs 5 and 6. Among the fractions, F10 (EA: MET-9:1) showed highest inhibitory activity (93 and 88%) against AChE and BuChE, but similar when compared to positive control donepezil (97 and 91% respectively).

#### LC-MS/MS analysis for identification of active fraction of ACTPG

**Fig 7:**
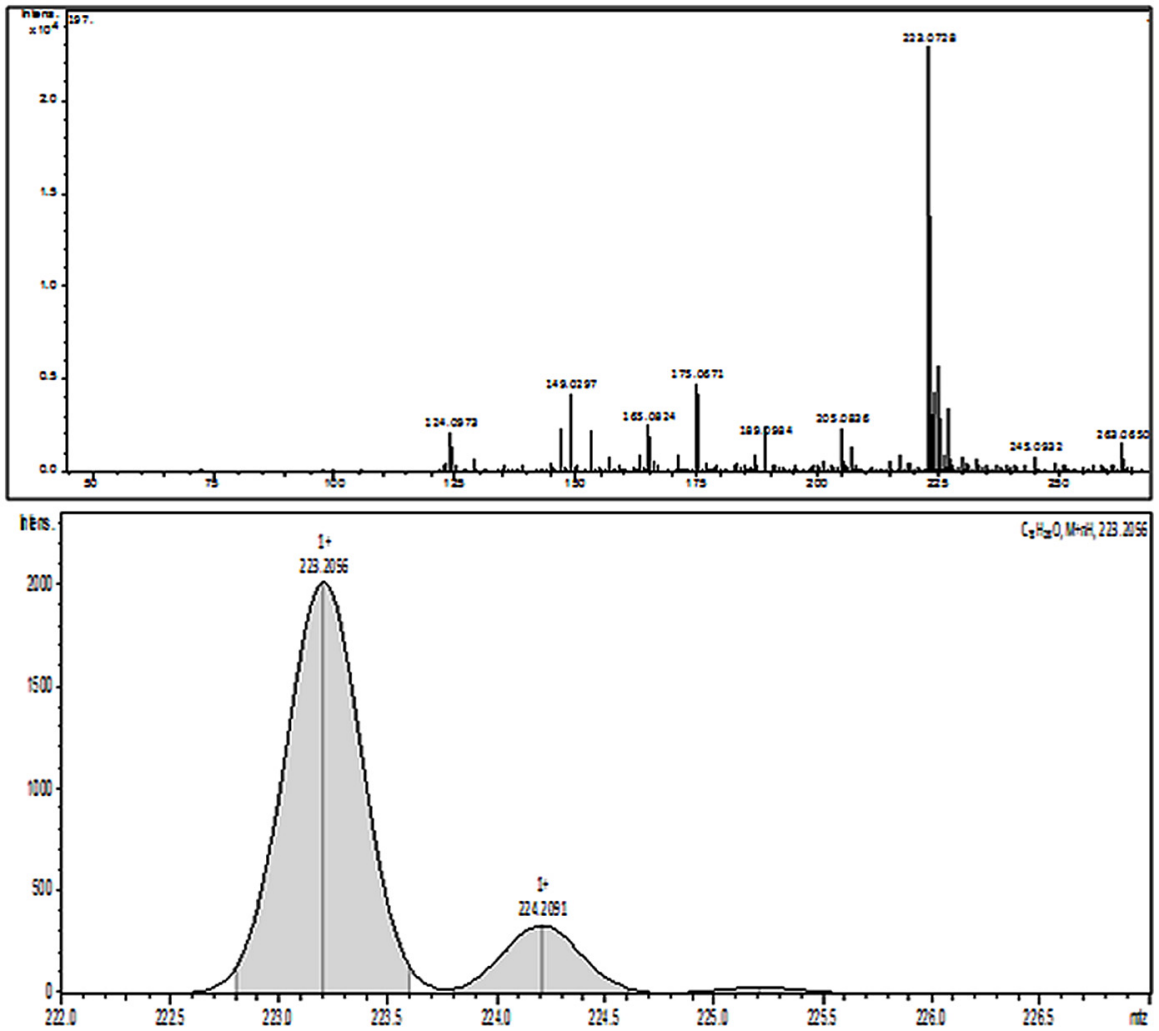
LC-MS/MS spectrum of active fraction (F10) of ACTPG. LC-MS/MS spectrum of active F10 fraction of ACTPG showed the presence of essential oil alpha-bisabolol.

Bioactivity guided fractionation of ACTPG was carried out to isolate and identify the active compound responsible for the neuroprotective effect. Recent report on GC-MS analysis of ethylacetate extract of *P*. *gymnospora* showed the presence of fucosterol as one of the major component. The identified compound fucosterol has been observed to possess several biological properties including antioxidant, cytotoxic, antidiabetic and acetylcholinesterase (ACE) inhibitory activities [30]. The present study primarily focused on the detection of anti-amyloidogenic agents from the marine brown seaweed *P*. *gymnospora*. The *in vitro* anti-cholinesterase assay was employed to screen the bioactivity for collected fractionations. Among the 18 fractions, fraction eluted with Ethyl acetate: Methanol (9:1) showed highest cholinergic inhibitory effect, which was subjected to LC-MS/MS analysis. The identity of the compound and its molecular mass was confirmed by comparing their m/z ratio with those on the stored library and the results are tabulated in Table 1. LC-MS/MS analysis (Fig 7) showed the presence of dipeptide L-Carnosine, essential oil alpha-bisabolol, narcotic pain reliever Propoxyphene, central nervous system (CNS) stimulant Caffeine, plasticizer Dibutyl phthalate, herbicide Foramsulfuron and a human metabolite D-mannitol 1-phosphate in active fraction of ACTPG. Among the different compounds, essential oil alpha-bisabolol has been reported for several pharmacological properties ranging from anti-inflammatory to anticancer activities and also reported to restore the redox balance by antagonising oxidative stress [31]. The bioassay was carried out to screen the bioactive compounds present in ACTPG such as L-Carnosine, Caffeine, and alpha-bisabolol for evaluating its neuroprotective effect.

**Table 1:** LC-MS/MS profile of ACTPG showing the presence of bioactive compounds.

SL. NO	COMPOUND NAME	MOLECULAR MASS
1	L-Carnosine	227.18
2	Alpha bisabolol	223.07
3	Propoxyphene	340.25
4	Caffeine	192.12
5	Dibutyl phthalate	279.16
6	Foramsulfuron	453.35
7	D-mannitol 1-phosphate	263.06

#### Determination of ChE inhibitory property of compounds analyzed through LC-MS/MS

**Fig 8:**
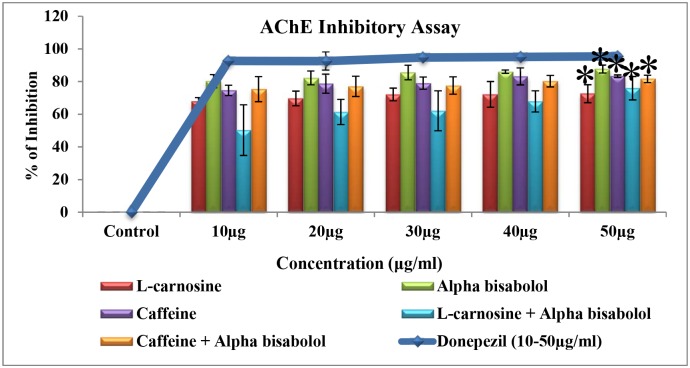
Acetylcholinesterase inhibitory activity of the active compounds (10–50 μg/ml). The result of AChE inhibitory activity clearly shows that among the different treatment groups, alpha-bisabolol alone treatment possessed a maximum AChE inhibitory activity(87%), with the IC50 value <10 μg/ml and showed significant inhibition (*p<0.1) at 50 μg/ml. Further, standard drug donepezil (IC50 value <6 μg/ml) and the compound of our interest, alpha-bisabolol displayed a significant inhibition on AChE. Values are expressed as Mean ± SD (n = 3).

**Fig 9:**
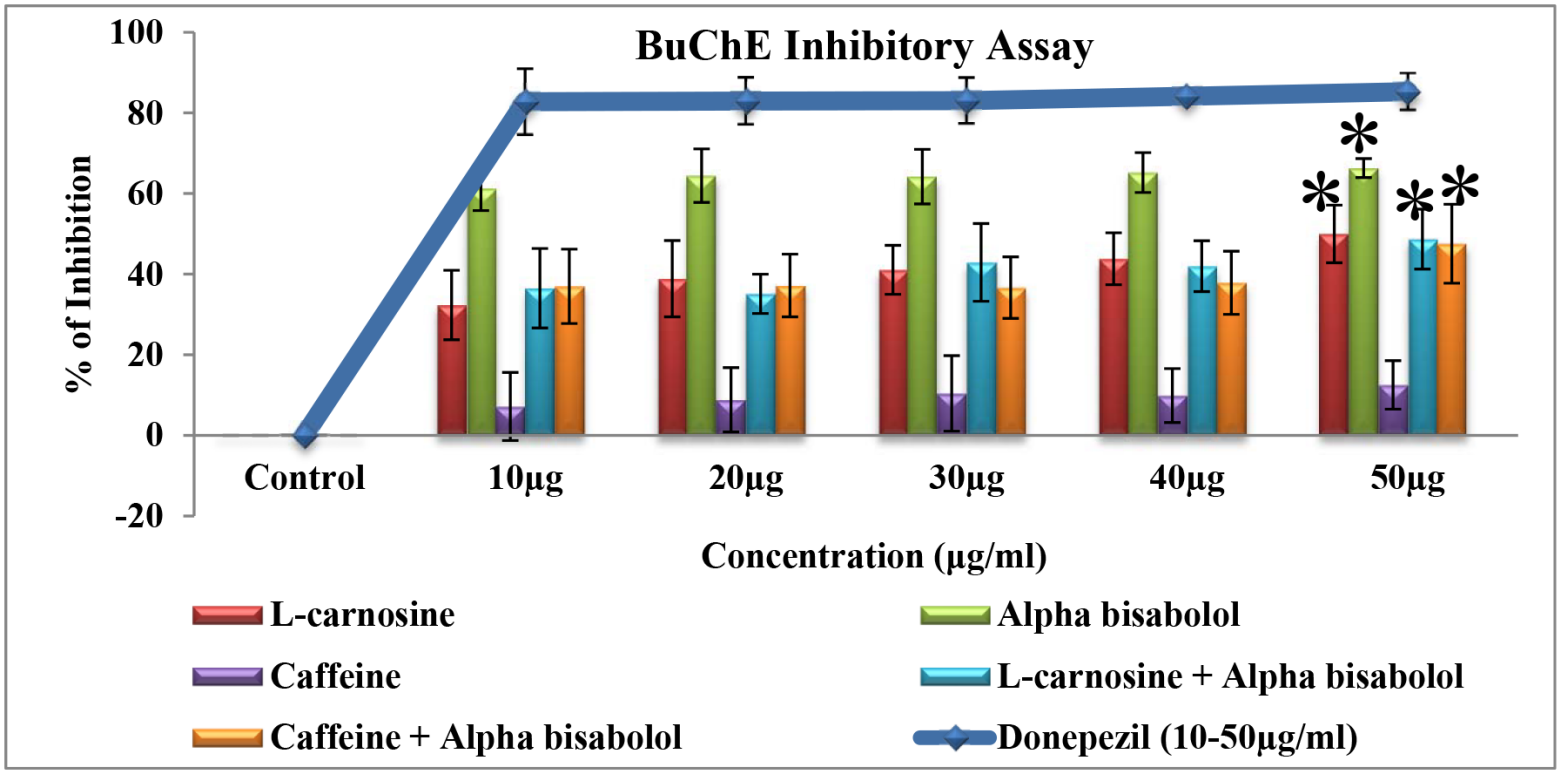
Butyrylcholinesterase inhibitory activity of the active compounds (10–50 μg/ml). The result of BuChE inhibitory activity clearly shows that among the different treatment groups, alpha-bisabolol alone treatment possessed a maximum BuChE inhibitory activity(66%), with the IC50 value <10 μg/ml and showed significant inhibition (*p<0.1) at 50 μg/ml. Further, standard drug donepezil (IC50 value <6 μg/ml) and the compound of our interest, alpha-bisabolol displayed a significant inhibition on BuChE. Values are expressed as Mean ± SD (n = 3).

AChE is also involved in secondary cholinergic functions by promoting Aβ (amyloid-beta) deposition in the form of senile plaque or neurofibrillary tangles, which is considered to be an important hallmark for the progression of AD [32]. Inhibition of AChE not only plays a key role in enhancing the cholinergic neurotransmission in the brain, but also aids in reducing the aggregation of β-amyloid, the key factor in AD. On the other hand, the enzyme butyrylcholinesterase (BuChE) acts as a co-regulator of cholinergic neurotransmitter by hydrolyzing ACh in synaptic cleft [33]. Level of BuChE in AD brain was found to be elevated by two fold, which in turn enhanced the neurotoxicity of neuritic plaques [34]. Hence inhibition of AChE and BuChE is considered as a promising approach for the treatment of AD. The results (Figs 8 and 9) indicate that, among the different treatment groups [treated with the compounds L-carnosine, caffeine, alpha-bisabolol alone or in combinations of alpha bisabolol & L-carnosine (1:1) and alpha bisabolol & caffeine (1:1)], alpha-bisabolol alone treatment possessed a maximum cholinergic inhibitory activity, with the IC50 value < 10 μg/ml for both AChE(87%) and BuChE(66%) and showed significant inhibition for both AChE and BuChE (p<0.1) at 50 μg/ml respectively. Further, standard drug donepezil (IC50 value < 6 μg/ml) and the compound of our interest, alpha-bisabolol displayed a significant inhibition on ChE when compared to the previously reported compounds L-Carnosine and Caffeine [35, 36]. Inhibition of AChE and BuChE plays a major role in reducing the aggregation pattern of β-amyloid, which is an important key factor in AD pathology. The present study clearly indicates that alpha-bisabolol possibly plays a substantial role by modulating the amyloidogenic properties of Aβ. Finally we conclude that, alpha-bisabolol could be used as a potent anti-amyloidogenic drug for AD treatment.

## Conclusion

In the present study, we have attempted to isolate multipotent drug from *P*. *gymnospora* to combat AD. Initial study was carried out using cell free *in vitro* system including antiaggregation and disaggregation assays of Aβ peptide. The results of our anti-Aβ aggregation studies confirm that ACTPG exhibits anti-amyloidogenic effects. Since ACTPG acts as multifunctional agent exhibiting antiaggregation and disaggregation of Aβ fibrils, it can act as a potent drug to combat the multiple targets of AD. The most probable reason for their potential of antiaggregation property might be related to the presence of alpha bisabolol. Thus, the current study for the first of its kind unveils the neuroprotective potential of alpha-bisabolol from *P*. *gymnospora*, as well as its suitability as a novel and alternative bioactive molecule to combat AD. Further studies on alpha-bisabolol are underway to decipher their anti-amyloidogenic property against AD.
